# Polyunsaturated Fatty Acid (PUFA) Composition of Growth Medium Changes the Atherogenic Potential of Human Aortic Endothelial Cells (HAECs) Following Endotoxin Stimulation

**DOI:** 10.3390/biomedicines13112706

**Published:** 2025-11-04

**Authors:** Nikolina Kolobarić, Zrinka Mihaljević, Mirjana Suver Stević, Ana Marinčić Žagar, Sandor G. Vari, Ines Drenjančević

**Affiliations:** 1 Department of Physiology and Immunology, Faculty of Medicine Osijek, J. J. Strossmayer University of Osijek, J. Huttlera 4, 31000 Osijek, Croatia; nbdujmusic@mefos.hr (N.K.); zmihaljevic@mefos.hr (Z.M.); amarincic@mefos.hr (A.M.Ž.); 2 Laboratory of Molecular and HLA Diagnostics, Department of Laboratory Diagnostics and Clinical Transfusion Medicine, Clinical Institute for Transfusion Medicine, Osijek University Hospital Center, J. Huttlera 4, 31000 Osijek, Croatia; suver-stevic.mirjana@kbco.hr; 3 International Research and Innovation in Medicine Program, Cedars-Sinai Medical Center, Los Angeles, CA 90089, USA

**Keywords:** cell adhesion molecules, fatty acids, lipopolysaccharides, ROS

## Abstract

**Background/Objectives:** Endothelial activation by lipopolysaccharides (LPS) contributes to inflammation and the development of cardiovascular disease, making *n-3* polyunsaturated fatty acids (PUFAs) potential modulators capable of mitigating endothelial dysfunction. The current study examines the effects of long-chain eicosapentaenoic acid (EPA) and docosahexaenoic acid (DHA), along with their precursor, α-linolenic acid (ALA), on oxidative stress, adhesion molecule expression, and cytokine milieu in LPS-stimulated human aortic endothelial cells (HAECs). **Methods:** HAECs (fifth passage) were cultured in control medium under standard conditions: ~37 °C, 5% CO_2_, ≥80% humidity. Cells were incubated in control basal cell medium or medium supplemented with ALA, EPA, DHA, and their combination (50 µM; *n* = 5 per group). After 48 h, cells were treated overnight (~16 h) with LPS from *E. coli* (0.75 and 1 µg/mL). HAECs and supernatants were collected for flow cytometry, Luminex, and ELISA assays. Significance was assessed using two-way analysis of variance ANOVA, followed by post hoc analyses (*p* < 0.05). Spearman’s correlation analysis was performed between markers, and *p*-values were adjusted using the Benjamini–Hochberg (BH) correction. **Results:** PUFA supplementation, particularly with DHA and ALA, significantly reduced intracellular reactive oxygen species (ROS) production and the expression of adhesion molecules (ICAM-1, E-selectin) in HAECs under both basal and LPS-stimulated inflammatory conditions. All PUFAs reduced pro-inflammatory cytokine levels (IFNγ, TNFα, IL-6), while ALA increased IL-1α and endoglin expression, indicating differential immunomodulatory effects. EPA exhibited antioxidant and anti-inflammatory effects primarily at higher LPS concentrations. Correlation analysis demonstrated strong interdependence between oxidative stress, inflammatory markers, and vascular activation, further confirming PUFA-mediated endothelial protection. **Conclusions:** PUFA supplementation produced molecule-specific effects on endothelial inflammation. DHA and ALA consistently showed anti-inflammatory and antioxidative effects, while EPA’s beneficial effect was more pronounced under inflammatory conditions, emphasising the importance of PUFA type and context in managing vascular inflammation.

## 1. Introduction

Long-chain polyunsaturated fatty acids (PUFAs) are metabolized from essential fatty acid (FA) precursors, linoleic acid (LA) and α-linolenic acid (ALA), through the actions of elongase and desaturase enzymes [1,2,3]. The availability of substrate determines whether these enzymes direct metabolism towards the *n-3* or *n-6* PUFA pathway. These pathways have distinct physiological roles. The *n-3* PUFA pathway primarily generates anti-inflammatory eicosapentaenoic acid (EPA) and docosahexaenoic acid (DHA), whereas the *n-6* PUFA pathway produces arachidonic acid (AA) and its mainly pro-inflammatory eicosanoid derivatives. Notably, certain *n-6*-derived metabolites, such as lipoxins, also exhibit anti-inflammatory and pro-resolving properties [3,4,5,6,7,8]. 

ALA supplementation has been shown to exert anti-hypertensive effects and to improve endothelial function. Specifically, it enhances endothelium-dependent vasorelaxation evoked by acetylcholine (ACh), attenuates reactive oxygen species (ROS) production, and decreases P-selectin and intercellular adhesion molecule 1 (ICAM-1) expression in spontaneously hypertensive rats (SHRs), high-fat diet-fed streptozotocin-induced (HFD-STZ) rats, and endothelial cell (EC) cultures [9,10,11]. Furthermore, ALA inhibits mitochondrial ROS overproduction and superoxide dismutase 2 (SOD2) hyperacetylation, and reduces the production of Vascular Endothelial Growth Factor (VEGF), regulated upon activation normal T cell expressed and secreted (RANTES), intercellular adhesion molecule 1 (ICAM-1), monocyte chemoattractant protein-1 (MCP-1), and interleukin 6 (IL-6) following tumour necrosis factor-alpha (TNF-α) stimulation in endothelial cells (ECs) [9,12]. An ALA-enriched diet has been shown to improve flow-mediated dilation (FMD) and reduce total and low-density lipoprotein (LDL)-cholesterol serum levels, C-reactive protein (CRP), E-selectin serum, and soluble vascular cell adhesion molecule (sVCAM) plasma levels in overweight and hypercholesterolaemic individuals [13,14,15]. Cross-sectional studies on healthy participants have shown a strong correlation between serum FA content, specifically ALA levels, and endothelial vasodilatory function, suggesting its potential as a predictor of atherosclerosis, particularly in young men [16,17].

Although dietary ALA is the precursor to more potent *n-3* PUFAs, its conversion to EPA, and especially to DHA, is fairly limited [8,18,19]. Direct EPA and/or DHA supplementation has been shown to decrease endotoxin-induced monocyte chemoattractant protein-1 (MCP-1) expression and nuclear factor-kappa B (NF-κB) activation in human kidney-2 cells (HK-2) [20]. It also increases uncoupling protein 3 (UCP3) gene expression in C2C12 muscle cells [21], improves endothelial function, decreases oxidative stress and blood pressure, and enhances high-density lipoprotein (HDL)-cholesterol in cardiometabolic patients [22,23,24,25]. Supplementation with these *n-3* PUFAs in the form of functional food improved ACh-induced dilation, decreased pro-inflammatory IL-6 and interferon gamma (IFNγ), and increased anti-inflammatory IL-10 levels in young healthy participants following a three-week dietary protocol [26,27]. In cardiovascular patients, the same dietary protocol decreased serum LDL cholesterol, hsCRP, IL-6, and IL-1, while in competitive athletes it resulted in enhanced microvascular function in response to vascular occlusion and ACh administration [28,29]. Recent clinical findings point to important differences between monotherapy and combined PUFA formulations in cardiovascular outcomes. While EPA treatment alone has yielded consistently significant improvements, combinations with other PUFAs have produced largely neutral results [30,31,32].

Despite these findings, it remains unclear how different *n-3* PUFAs affect endothelial activation at the cellular level in response to inflammatory stimuli, such as lipopolysaccharides (LPS), which are potent activators of endothelial cells through toll-like receptor 4 (TLR4), NF-κB, and mitogen-activated protein kinase (MAPK) pathways, inducing ROS production, cytokine release, and adhesion molecule expression [33,34,35,36,37,38,39,40,41,42]. 

In the present study, we used human aortic endothelial cells (HAECs) to investigate the effects of *n-3* PUFAs on ROS production, adhesion molecule expression, cytokine secretion, and NF-κB signalling following LPS stimulation. We hypothesise that *n-3* PUFA treatment will attenuate LPS-induced endothelial activation in a PUFA type-dependent manner.

## 2. Materials and Methods

### 2.1. Materials and Chemical Reagents

HAECs were purchased from Innoprot (Barcelona, Spain). Cell culture flasks and well-plates were purchased from TPP Techno Plastic Products AG (Trasadingen, Switzerland). Human Large Vessel Endothelial Cell Basal Medium, low serum growth supplement (LSGS) and Trypsin-EDTA (0.25%) were purchased from Gibco (Thermo Fisher Scientific, Waltham, MA, USA). Lipopolysaccharides (LPS) from *E. coli* and PUFAs ALA (C18:3, *n*-3), EPA (C20:5, *n*-3) and DHA (C22:6, *n*-3) were purchased from Sigma Aldrich (Merck Group, Darmstadt, Germany). Flow cytometry antibodies for ICAM-1 (CD54, clone: 15.2) were purchased from Proteintech Group Inc. (Rosemont, IL, USA), while antibodies to VCAM-1 (CD106, clone: 51-10C9), E-selectin (CD62E, clone: 68-5H11) and endoglin (CD105, clone: 266) were purchased from BD Pharmingen Inc. (Franklin Lakes, NJ, USA). Fixable viability dye (FVD) eFluor™ 780 was purchased from eBioscience^TM^ (Invitrogen by Thermo Fisher Scientific, Waltham, MA, USA). Invitrogen Human ProcartaPlex Mix & Match 5-plex kit for quantification of IFNγ, IL-1α, IL-23, IL-6 and TNFα was purchased from Invitrogen (Thermo Fisher Scientific, Waltham, MA, USA). NF-κB p65 ELISA kit was purchased from Abcam (Cambridge, UK).

### 2.2. HAECs: Cell Culture Treatment 

The fourth passage (P4) of HAECs was quickly thawed from liquid nitrogen, seeded in adherent cell culture flasks (T-25 cm^2^), and grown in an incubator (Shel Lab, CO_2_ Series, Sheldon Manufacturing Inc., Cornelius, OR, USA) under standard conditions (37 °C, 5% CO_2_, >80% humidity). Human Large Vessel Endothelial Cell Basal Medium supplemented with LSGS (M200) was used throughout the experiment. HAECs were monitored daily, and the medium was changed every 2–3 days until confluence was achieved. The fifth passage (P5) of cells was used at approximately 80% confluence for all experiments. A short trypsinization method was used to detach HAECs from the flask/plate surface.

**Figure 1:**
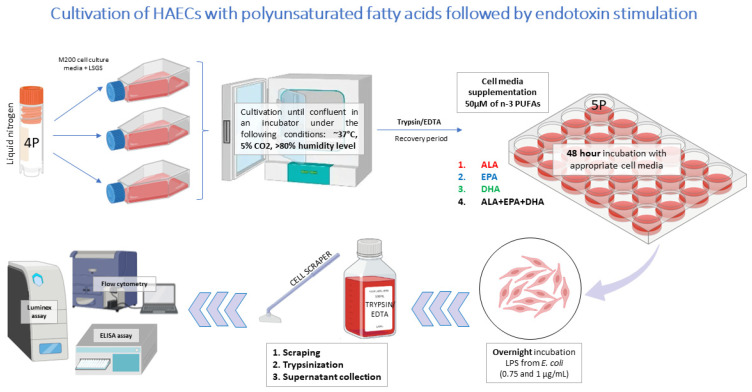
Study design. EPA—eicosapentaenoic acid; DHA—docosahexaenoic acid; ALA—alpha linolenic acid; PUFA—polyunsaturated fatty acid; LPS—lipopolysaccharides.

The study design is shown in Figure 1. After trypsinisation, HAECs (P5) were transferred to 24- or 96-well plates, depending on the subsequent experiments. After 24 h, basal cell media was replaced with different PUFA-supplemented media (50 µM) for an additional 48 h. The 50 µM PUFA concentration was selected based on preliminary optimisation and previous reports demonstrating effective yet non-toxic ranges in endothelial and other cell types [43,44,45]. Higher concentrations (≥75 µM) resulted in loss of cell attachment and viability in our preliminary experiments. On the final day of PUFA incubation, LPS was added overnight (16 h). Cells were stimulated with two different concentrations of LPS, 0.75 and 1 µg/mL [46,47].

Experimental groups included in the study were:

1. Basal cell medium group (control);
2. ALA-supplemented cell medium group (ALA);
3. EPA-supplemented cell medium group (EPA);
4. DHA-supplemented cell medium group (DHA);
5. ALA/EPA/DHA-supplemented cell medium group (3PUFA).

Within each experimental group, three subgroups were designated based on LPS stimulation and its concentration:

(a) No stimulation—baseline conditions;
(b) Stimulation with 0.75 µg/mL of LPS (LPS75)—moderate inflammatory conditions;
(c) Stimulation with 1 µg/mL of LPS (LPS100)—high inflammatory conditions.

All experiments were conducted using five independent experimental replicates. For each experimental replicate, cells were thawed from a separate cryotube of the same purchased cell line, ensuring an independently cultured population for each replicate. Within each experimental replicate, four technical replicates (multiple wells per treatment) were used to assess intra-assay variability and ensure consistency of measurements.

### 2.3. Flow Cytometry

Samples were collected after 48 h exposure of HAECs to different PUFAs and overnight stimulation with LPS. Cells were washed in 1× phosphate-buffered saline (PBS) and prepared for staining (approximately 10^6^ cells/mL). FACS Canto II flow cytometer (BD Bioscience; 488 excitation laser and 530/30 BP analysis filter) was used to assess intracellular ROS production and CAM expression. Cell viability was assessed by staining the cells with fixable viability dye (FVD) eFluor™ 780 (eBioscienceTM, Invitrogen by Thermo Fisher Scientific, Waltham, MA, USA). Data analysis and visualisation were performed using Flow Logic software v.8 (Inivai Technologies, Mentone, Australia). 

#### 2.3.1. Intracellular ROS Production

Dichlorofluorescein diacetate (DCF-DA) was used to determine the levels of hydrogen peroxide (H_2_O_2_) and peroxynitrite (ONOO^−^), while dihydroethidium (DHE) was used to determine the level of superoxide anion (O_2_^−^). The principle of the assay was previously described in detail [48]. Cells were re-suspended 1× PBS and incubated with both fluorescent dyes (10 µM final concentration). Samples stained with DCF-DA were resuspended in 1× PBS and immediately assessed on a cytometer. Samples stained with DHE required additional rinsing before resuspension in 1× PBS and cytometer analysis. Fluorescence intensity is presented as the geometric mean (GMFI) for the FLH-1 and FLH-2 channels.

#### 2.3.2. CAM Expression

Cells were resuspended and washed twice in 1× PBS. Before staining with the appropriate antibody mixture, cell viability was assessed by staining with FVD, which irreversibly labels dead cells. For assessment of CAM expression, HAECs were stained with anti-CD54 PE (ICAM-1, clone: 15.2), anti-CD106 FITC (VCAM-1, clone: 51-10C9), anti-CD62E APC (E-selectin, clone: 68-5H11) and anti-CD105 PerCP-Cy5 (endoglin, clone: 266). Appropriate controls were included in the final analysis. Data was presented as GMFI. A representative gating strategy is shown in the Supplementary Materials (Figure S1: Representative gating).

### 2.4. Luminex Assay

A luminex 5-plex kit with a custom-blended panel was used to measure the concentrations of IFNγ, IL-1α, IL-23, IL-6 and TNFα in cell culture supernatants (Invitrogen by Thermo Fisher Scientific, Waltham, MA, USA). Supernatants were collected after the designated incubation, before trypsinisation, and stored at −80 °C until use. The assay was performed as recommended in the user guide provided with the kit. Measurements were conducted on the Luminex 200 instrument platform (Luminex Corp., Austin, TX, USA) in the Laboratory of Molecular and HLA Diagnostics at the University Hospital Osijek (Osijek, Croatia). Data analysis was performed using ProcartaPlex Analyst free software (eBioscience, Affymetrix by Thermo Fisher Scientific, Waltham, MA, USA). Concentrations are expressed in picograms per millilitre (pg/mL).

### 2.5. ELISA Assay

Semi-quantitative measurement of total NF-κB p65 in cell lysates was performed using a commercially available enzyme-linked immunosorbent assay (Abcam, Cambridge, UK). Well optical density (OD) was recorded on a microplate reader (BioRad PR 3100 TSC, Bio-Rad Laboratories, CA, USA) at 450 nm. Cell lysates were prepared as advised in the provided protocol for adherent cells. Approximately, 30,000 HAECs were solubilised by the addition of cell extraction buffer. Samples were stored at −80 °C until required for the assay.

### 2.6. Statistical Analysis

Sample size was calculated using GPower 3.1 (Heinrich Heine University Düsseldorf, Düsseldorf, Germany), and it was estimated that at least four samples per group were necessary to achieve a power of 0.80 at a significance level of 0.05, assuming a large effect size (0.50). This effect size was selected based on preliminary data. The normality of the residuals, where appropriate, was assessed using the Shapiro–Wilk test, while the homogeneity of variances was determined using the Brown–Forsythe test. Significant differences between groups and interactions were determined using two-way ANOVA followed by Tukey’s test (for comparisons between different PUFA treatments within each LPS condition) or Dunnett’s test (for comparisons of LPS-stimulated groups with the non-stimulated control within each PUFA treatment) multiple comparisons. Sidak’s correction was applied where appropriate. Spearman’s rank correlation analysis was performed to assess potential associations between inflammatory and endothelial markers within each treatment group. The Benjamini–Hochberg (BH) correction was applied to control the false discovery rate. All statistical analyses were performed using Microsoft Excel 2021 (Microsoft® Excel® LTSC MSO 64-bit, Microsoft Corporation, Redmond, WA, USA) and Graph Pad Prism v8 (GraphPad Software, San Diego, CA, USA).

## 3. Results

### 3.1. Flow Cytometry

#### 3.1.1. Intracellular ROS Production in HAECs: DCF-DA and DHE Assay

**Figure 2:**
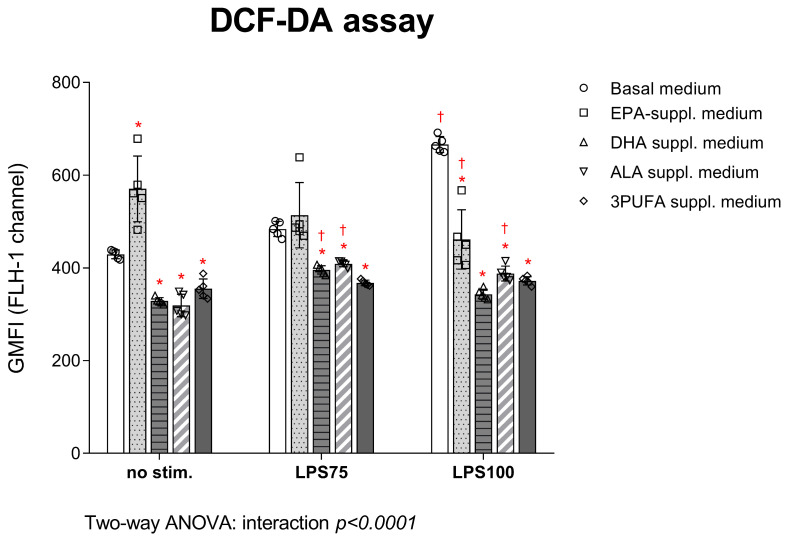
Intracellular formation of hydrogen peroxide and peroxynitrite in HAECs supplemented with *n-3* PUFAs followed by LPS exposure. Results are expressed as geometric mean fluorescence intensity (GMFI). DCF-DA—dichlorofluorescein diacetate; HAEC—human aortic endothelial cells; EPA—eicosapentaenoic acid; DHA—docosahexaenoic acid; ALA—alpha linolenic acid; PUFA—polyunsaturated fatty acid; LPS—lipopolysaccharides. Two-way ANOVA; significance level *p* < 0.05 (* compared to control group with basal cell medium; † compared to baseline conditions without LPS stimulation).

DCF-DA assay results are presented in Figure 2. Following supplementation of the cell medium with DHA, ALA, and 3PUFA, intracellular H_2_O_2_ and ONO^−^ production in HAECs significantly decreased compared to the control group, both before (DHA *p* = 0.001; ALA *p* = 0.0006; 3PUFA *p* = 0.02) and after stimulation with LPS at both concentrations (LPS75: DHA *p* = 0.004; ALA *p* = 0.015; 3PUFA *p* = 0.0001; LPS100: DHA *p* < 0.0001; ALA *p* < 0.0001; 3PUFA *p* < 0.0001). Following EPA supplementation, a significant decrease in these ROS productions was observed only after stimulation with the highest LPS concentration (*p* < 0.0001). Intra-group analysis indicated a significant decrease in intracellular H_2_O_2_ and ONOO^−^ production following stimulation with the higher LPS concentration in the EPA-supplemented group (*p* = 0.0007), while there was a slight increase in DHA (*p* = 0.03)- and ALA (*p* = 0.006)-supplemented HAECs compared to baseline conditions. 

**Figure 3:**
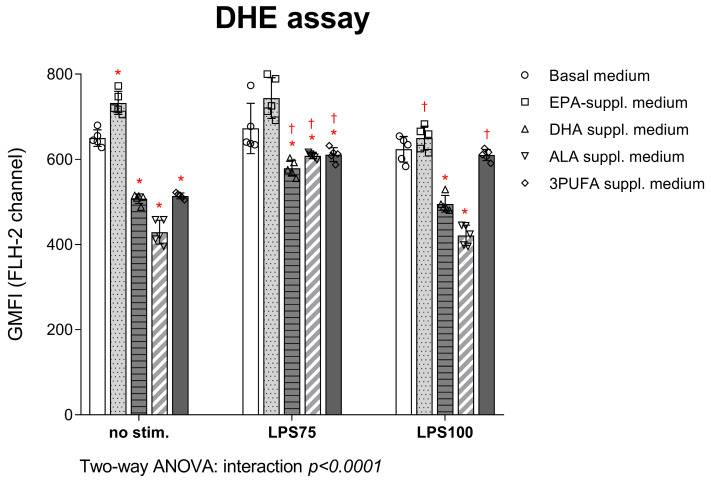
Intracellular formation of superoxide in HAECs supplemented with *n-3* PUFAs followed by LPS exposure. Results are expressed as geometric mean fluorescence intensity (GMFI). DHE—dihydroethidium; HAEC—human aortic endothelial cells; EPA—eicosapentaenoic acid; DHA—docosahexaenoic acid; ALA—alpha linolenic acid; PUFA—polyunsaturated fatty acid; LPS—lipopolysaccharides. Two-way ANOVA; significance level *p* < 0.05 (* compared to control group with basal cell medium; † compared to baseline conditions without LPS stimulation).

DHE assay results are shown in Figure 3. O_2_^−^ production was significantly reduced following DHA and ALA supplementation, both before (DHA *p* < 0.0001; ALA *p* < 0.0001) and after LPS stimulation, at both concentrations (LPS75: DHA *p* < 0.0001; ALA *p* = 0.004; 3PUFA *p* = 0.006; LPS100: DHA *p* < 0.0001; ALA *p* < 0.0001). EPA supplementation did not significantly alter ROS production after stimulation compared to basal cell medium. Supplementation with all three *n-3* PUFAs led to decreased ROS production both before (*p* < 0.0001) and after lower concentration LPS stimulation (*p* = 0.006). Intra-group analysis showed a significant decrease in intracellular O_2_^−^ production in EPA-supplemented HAECs following stimulation under high inflammatory conditions (*p* = 0.001), while it increased in the DHA (*p* = 0.003), ALA (*p* < 0.0001), and 3PUFA (*p* < 0.0001) groups following stimulation under moderate inflammatory conditions. Intracellular ROS production significantly decreased in DHA (*p* = 0.0007)- and ALA (*p* < 0.0001)-supplemented HAECs following high inflammation compared to moderate inflammation stimulation, returning production close to baseline conditions.

#### 3.1.2. Expression of CAMs in HAECs

**Figure 4:**
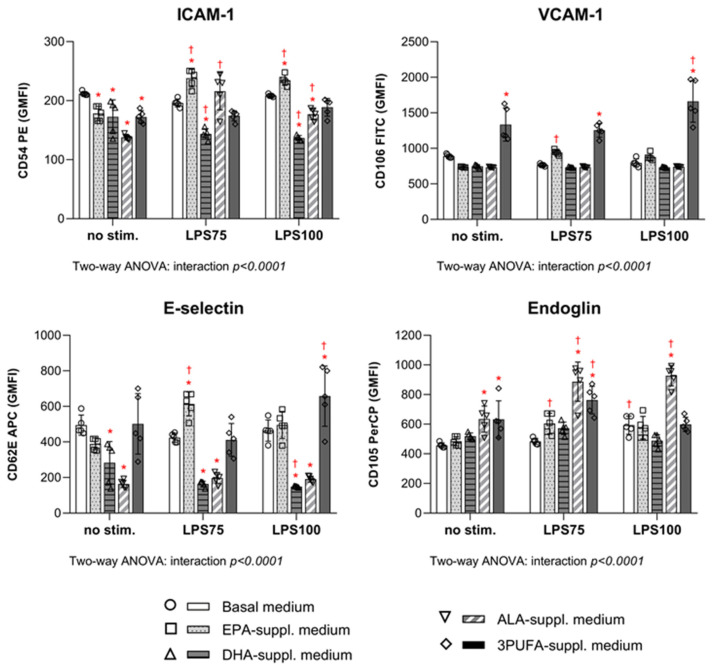
CAM expression in HAECs following supplementation with *n-3* PUFAs followed by LPS from *E. coli* exposure. Results are expressed as geometric mean fluorescence intensity (GMFI). CAMs—cell adhesion molecules; HAECs—human aortic endothelial cells; EPA—eicosapentaenoic acid; DHA—docosahexaenoic acid; ALA—alpha linolenic acid; PUFA—polyunsaturated fatty acid; LPS—lipopolysaccharides. Two-way ANOVA; significance level *p* < 0.05 (* compared to control group with basal cell medium; † compared to baseline conditions without LPS stimulation).

The effects of PUFA supplementation on CAM expression in HAECs before and after LPS stimulation are shown in Figure 4. Treatment with DHA and ALA resulted in a significant decrease in ICAM-1 and E-selectin expression both prior to (for ICAM-1, DHA *p* = 0.0004, ALA *p* < 0.0001; for E-selectin, DHA *p* = 0.001, ALA *p* < 0.0001) and following LPS stimulation (for ICAM-1, DHA *p* < 0.0001, ALA *p =* 0.006; for E-selectin, DHA *p* < 0.0001, ALA *p* < 0.0001) in HAECs compared to the control group. However, ALA supplementation caused a significant increase in endoglin expression under all experimental conditions (no stim. *P* = 0.002; LPS75 *p* < 0.0001; LPS100 *p* < 0.0001). EPA supplementation led to a significant reduction in ICAM-1 expression before stimulation (*p* = 0.003), but a significant increase following endotoxin stimulation (LPS75 *p* = 0.0001; LPS100 *p* = 0.036). VCAM-1 expression was largely unaffected by individual PUFAs, while combined PUFA treatment significantly increased expression both in the absence (*p* < 0.0001) and presence of LPS (LPS75 *p* < 0.0001; LPS100 *p* < 0.0001).

In response to endotoxin exposure, intra-group analysis showed that LPS stimulation predominantly affected ICAM-1 and endoglin expression among PUFA-treated groups, significantly increasing their levels under both moderate (ICAM-1: EPA *p* < 0.0001, ALA *p* < 0.0001; endoglin: EPA *p* = 0.03, ALA *p* < 0.0001, 3PUFA *p* = 0.02) and high inflammatory conditions (ICAM-1: ALA *p* = 0.0001; endoglin: ALA *p* < 0.0001) compared to baseline. However, there was a significant decrease in E-selectin and ICAM-1 expression in DHA-supplemented HAECs following inflammatory stimulation (E-selectin: LPS100 *p* = 0.03; ICAM-1: LPS75 *p* = 0.005, LPS100 *p* = 0.0004). Furthermore, under high inflammatory conditions (LPS100), only VCAM-1 and E-selectin expression were significantly increased in 3PUFA-supplemented HAECs. Most of the other CAM expressions did not show significant changes in response to the varying LPS concentrations. Cell viability remained above 80% in the parent population across all experimental groups, with no significant changes before or after treatment or stimulation.

### 3.2. Endotoxin-Induced Cytokine Concentrations Following n-3 PUFA Treatment

**Figure 5:**
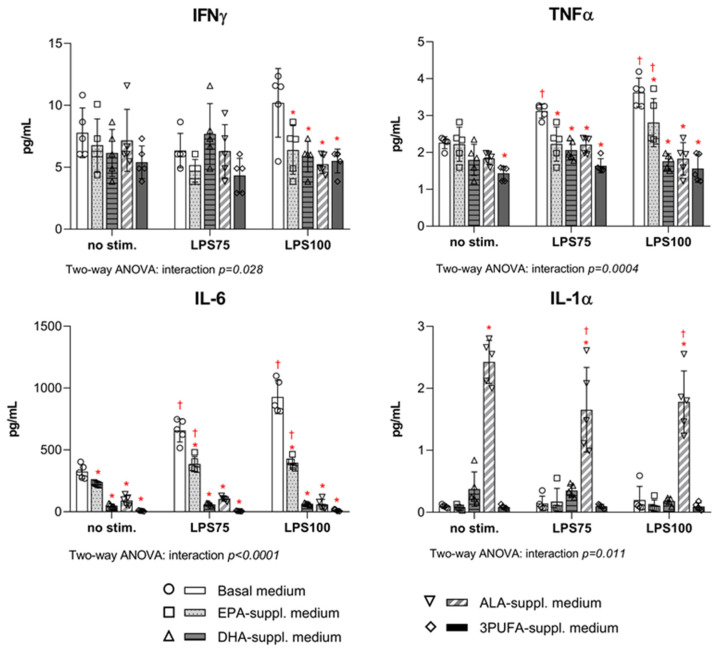
Levels of pro-inflammatory cytokines and chemokines secreted by HAECs upon LPS from *E. coli* stimulation following PUFA supplementation. HAEC—human aortic endothelial cells; LPS—lipopolysaccharides; IFNγ—interferon gamma; TNFα—tumour necrosis factor alpha; IL-6—interleukin 6; IL-1α—interleukin 1 alpha. Two-way ANOVA; significance level *p* < 0.05 (* compared to control group with basal cell medium; † compared to baseline conditions without LPS stimulation).

Pro-inflammatory cytokine (IFNγ, TNFα, IL-6, IL-1α) concentrations following PUFA supplementation and endotoxin stimulation were measured in HAEC supernatants (Figure 5). The IFNγ concentration was significantly reduced in all PUFA-supplemented groups after stimulation under high inflammatory conditions (LPS100) compared to baseline (EPA *p* = 0.002; DHA *p* = 0.006; ALA *p* = 0.0003; 3PUFA *p* = 0.0005). The TNFα supernatant concentration was significantly reduced after stimulation with both LPS concentrations in all PUFA-supplemented groups (LPS75: EPA *p* = 0.0006, DHA *p* = 0.0001, ALA *p* = 0.0005, 3PUFA *p* < 0.0001; LPS100: EPA *p* = 0.002, DHA *p* < 0.0001, ALA *p* < 0.0001, 3PUFA *p* < 0.0001). There was a significant decrease in supernatant IL-6 concentration in all experimental groups, both before (EPA *p* = 0.011, DHA *p* < 0.0001, ALA *p* < 0.0001, 3PUFA *p* < 0.0001) and after LPS stimulation (LPS75: EPA *p* < 0.0001, DHA *p* < 0.0001, ALA *p* < 0.0001, 3PUFA *p* < 0.0001; LPS100: EPA *p* < 0.0001, DHA *p* < 0.0001, ALA *p* < 0.0001, 3PUFA *p* < 0.0001). The IL-1α supernatant concentration was significantly increased in the ALA-supplemented group both before (*p* < 0.0001) and after LPS stimulation compared to the control group (LPS75: *p* < 0.0001; LPS100: *p* < 0.0001). IL-23 concentrations were undetectable in supernatant samples.

In the following intra-group analysis, TNFα and IL-6 supernatant concentrations were found to increase significantly in both basal and EPA-supplemented medium after exposure to both LPS75 (TNFα: no stim. *p* = 0.001; IL-6: no stim. *p* < 0.0001, EPA *p* < 0.0001) and LPS100 (TNFα: basal *p* < 0.0001, EPA *p* = 0.024; IL-6: no stim. *P* < 0.0001, EPA *p* < 0.0001) compared to baseline conditions.

### 3.3. Semi-Quantitative Measurement of NF-κB p65 in HAEC Lysates Following n-3 PUFA Treatment and Endotoxin Stimulation

**Figure 6:**
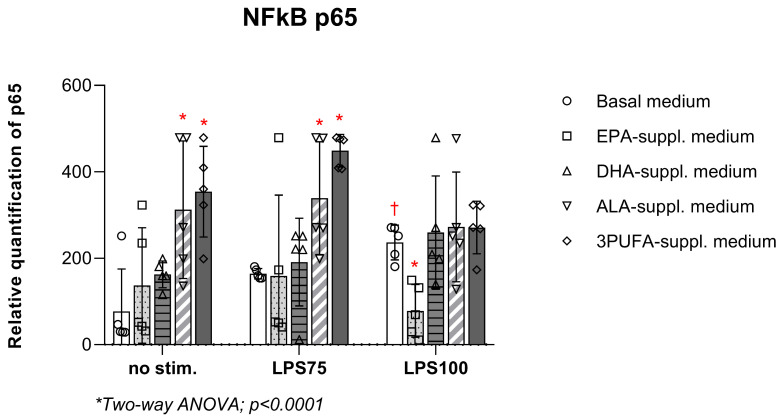
Relative quantification of NF-κB p65 protein in HAEC lysates upon LPS from *E. coli* stimulation following PUFA supplementation. HAECs—human aortic endothelial cells; LPS—lipopolysaccharides; NF-κB—nuclear factor kappa B. Two-way ANOVA; significance level *p* < 0.05 (* compared to control group with basal cell medium; † compared to baseline conditions without LPS stimulation).

Semi-quantitative analysis of p65 protein (NF-κB pathway) in HAEC lysates (Figure 6) showed that the endotoxin-induced increase observed in the control group (basal cell medium) (*p* = 0.04) was eliminated following PUFA treatment under high inflammatory conditions. There was a significant increase in the relative quantification of p65 following ALA supplementation (no stim. *p* = 0.004; LPS75 *p* = 0.041) and combined PUFA supplementation (no stim. *p* = 0.0005; LPS75 *p* = 0.0003), while this increase was lost under high inflammatory conditions. EPA supplementation significantly decreased p65 in HAEC lysates under high inflammatory conditions compared to the control group with basal cell medium (*p* = 0.001). No significant changes were detected in the remaining groups.

### 3.4. Correlation Analysis

Correlation analysis was conducted between oxidative stress, endothelial, and pro-inflammatory parameters to explore statistically significant relationships and identify patterns of their co-dependence under different treatment or stimulation conditions. A correlation matrix was generated for each treatment to assess potential PUFA-specific relationships among the measured parameters. The table with *p*-values following correction for the correlation matrix is provided in the Supplementary Materials (Table S1: Correlation matrix *p*-values).

In the control group (basal cell media), significant positive correlations were observed between endothelial marker expression and pro-inflammatory cytokine concentrations (endoglin vs. TNF-α *p* = 0.001, r = 0.781; endoglin vs. IL-6 *p* < 0.0001, r = 0.915) as well as among CAMs within each category (ICAM-1 vs. VCAM-1 *p* = 0.0009, r = 0.838; VCAM-1 vs E-selectin *p* = 0.0009, r = 0.849). Furthermore, both exhibited strong positive correlations with intracellular hydrogen peroxide and peroxynitrite production (endoglin vs. DCF-DA *p* = 0.0005, r = 0.835; TNF-α vs. DCF-DA *p* = 0.001, r = 0.796; IL-6 vs. DCF-DA *p* < 0.0001, r = 0.951). There was also a significant positive correlation between the relative quantification of NF-κB p56, IL-6 (*p* = 0.007, r = 0.743), and intracellular ROS production (DCF-DA *p* = 0.005, r = 0.783).

Following ALA supplementation, a significant negative correlation was observed between ICAM-1 and IL-1α supernatant concentration (*p* = 0.043, r = −0.706), while CAM expression also showed positive correlations with each other (ICAM-1 vs. endoglin *p* = 0.040, r = 0.711; ICAM-1 vs. E-selectin *p =* 0.003, r = 0.821). Intracellular ROS production positively correlated with ICAM-1 (*p* = 0.009, r = 0.784), and showed a significant negative correlation with IL-1α (*p* = 0.037, r = −0.659). EPA supplementation resulted in significant positive correlations of CAM expression, both mutually (VCAM-1 vs. ICAM-1 *p* = 0.007, r = 0.763; VCAM-1 vs. endoglin *p* = 0.014, r = 0.706; VCAM-1 vs. E-selectin *p* = 0.001, r = 0.846; ICAM-1 vs. endoglin *p* = 0.0015, r = 0.810; ICAM-1 vs. E-selectin *p =* 0.0018, r = 0.817; endoglin vs. E-selectin *p* = 0.002, r = 0.781) and with pro-inflammatory IL-6 supernatant concentration (VCAM-1 p = 0.042, r = 0.605; ICAM-1 *p* = 0.004, r = 0.745; E-selectin *p* = 0.036, r = 0.616). After DHA supplementation, there was a significant positive correlation between ICAM-1 and E-selectin expression (*p* = 0.001, r = 0.846), as well as between endoglin expression and IL-1α concentration (*p* = 0.03, r = 0.579). There was also a significant positive correlation between endoglin expression and TNFα concentration (*p* = 0.03, r = 0.674). Following combined PUFA supplementation, similar to the trends observed with EPA supplementation, vascular marker expression positively correlated among themselves (VCAM-1 vs. ICAM-1 *p* = 0.033, r = 0.654; VCAM-1 vs. E-selectin *p* = 0.045, r = 0.704) and with IL-6 concentration (VCAM-1 *p* = 0.026, r = 0.694). 

## 4. Discussion

Experimental studies using HAECs ensure that the obtained findings are directly relevant to human health, particularly for understanding the potential benefits of *n-3* PUFAs for managing cardiovascular health and inflammation. Noteworthy findings from the present study include the following: (1) *n-3* PUFA supplementation significantly decreased intracellular ROS production in HAECs under both moderate and high inflammatory conditions; (2) downregulation of pro-inflammatory cytokines following high inflammation in *n-3* PUFA-supplemented HAECs; (3) and dose-dependent modulation of immune cell adhesion and endothelial activation following *n-3* PUFA supplementation under inflammatory conditions.

Extensive scientific research over the past five decades has demonstrated the beneficial effects of *n-3* PUFAs on various health-related biomarkers, including oxidative stress, inflammation, micro- and macrocirculatory activity, and lipid profile [49,50,51,52]. Our research group previously reported that *n-3* PUFA-enriched functional foods reduced oxidative stress markers and pro-inflammatory cytokine concentrations in healthy individuals [26,27,53,54,55]. Further, it was previously reported that treatment with EPA, DHA, and ALA and their metabolites, followed by LPS stimulation, reduces pro-inflammatory cytokine production (TNF-α, IL-1β, IL-6) [56,57,58]. It also attenuates inflammatory responses through PPAR-γ activation and NF-κB inhibition, as previously reported in microglial cells [59,60], human kidney-2 (HK-2) cells (20), macrophages [61], endotoxic rats and mice [62,63,64,65,66]. These findings are consistent with the results of our current study, in which IFN-γ, TNF-α, and IL-6 supernatant concentrations significantly decreased following *n-3* PUFA supplementation under moderate and high inflammatory conditions. In the current study, PUFA supplementation significantly reduced intracellular production of H_2_O_2_, ONOO^−^ and O_2_^−^, prior to (DHA, ALA, 3PUFA groups) and following endotoxin stimulation (EPA, DHA, ALA, 3PUFA groups). Similar effects were observed in athletes and young healthy participants following dietary interventions with *n-3* PUFA-enriched chicken meat and eggs [53,54]. However, EPA supplementation resulted in a significant increase in ROS production in HAECs under baseline conditions, followed by a decrease under high inflammatory conditions. This initial mild oxidative stress can be explained as a by-product of EPA metabolism and its incorporation into cellular membranes. It may also reflect effects on mitochondrial function and the molecule‘s susceptibility to oxidation [67,68,69]. The subsequent decrease in ROS production following endotoxin stimulation supports EPA’s anti-inflammatory activity, potentially mediated through downregulation of the NF-κB pathway and effects on PPARs. This was also corroborated by our results, which indicated a significant decrease in p65 (NF-κB) expression under high inflammation exclusively in the EPA-supplemented group. Furthermore, relative quantification of p65 strongly correlated with key pro-inflammatory cytokine IL-6 as well as intracellular ROS production only in basal cell medium (control group), while these correlations were lost in *n-3* PUFA-supplemented groups.

CAM expression, on the other hand, exhibited a variety of changes following supplementation and endotoxin stimulation, corroborating the hypothesis that *n-3* PUFA supplementation modulates endothelial function through effects on CAMs. In the current study, DHA and ALA supplementation led to a significant downregulation of ICAM-1 and E-selectin expression in HAECs under higher inflammation, suggesting an anti-inflammatory effect on key adhesion molecules involved in leukocyte recruitment and interaction with ECs [70]. A study performed on human umbilical vein endothelial cells (HUVECs) concluded that the LPS-induced upregulation of ICAM-1 expression is time- and dose-dependent as well as mediated by the p38 MAPK pathway at the level of gene transcription [71]. Previous studies reported that a continuous exposure of endothelial cells to LPS, TNF-α, or IL-1β is required to elicit a maximal cellular response (E-selectin expression upregulation and NF-κB activation), with peaks between hours 4 and 6, returning to basal level by 24 h [72,73,74]. These might be the reasons for the absence of a significant effect on expression following exposure to LPS without PUFA in our study, due to shorter incubation. Nevertheless, Huang et al. [75] suggested inhibition of ICAM-1 and VCAM-1 expression due to reduced adhesion of THP-1 monocytes to HAECs in the presence of DHA and EPA [22]. Furthermore, DHA reduced the levels of E-selectin expressed on the surface of HUVECs following activation by TNF-α [76], while ALA inhibited endothelial inflammation in streptozotocin-induced diabetic rats and HUVECs through decreased P-selectin and ICAM-1 expression, inhibition of iNOS, and increased eNOS activity [10,11]. 

Interestingly, ALA supplementation significantly increased endoglin expression and IL-1α concentration across all conditions, suggesting a regulatory role of ALA in modulating endothelial activation and inflammatory signalling. Endoglin is a transmembrane receptor highly expressed on activated ECs. It has been described as either a suppressor or promoter of angiogenesis [77], a coreceptor in the TGFβ pathway [78], and a strong regulator of inflammatory response [79], EC proliferation [78], and behaviour [80,81,82,83,84]. Despite being a vascular marker, endoglin is not necessarily pro-inflammatory as it regulates other markers in inflammatory conditions. It was reported by Ruiz-Remolina et al. (2017) that human soluble endoglin employed in transgenic mice did not elicit any inflammation in control animals, while it led to down-regulation of pro-inflammatory cytokines TNF-α, IL-6, and IL-1β in induced inflammatory conditions [79]. ALA’s effects on endoglin may be indirect, potentially mediated through modulation of endothelial activation and inflammation through lipid mediators and activation of AMP-activated protein kinase (AMPK) [12,85,86,87]. Endoglin regulates the TGF-β signalling pathway, while activated AMPK inhibits it [78,88,89,90,91,92]. AMPK has been quoted as an important enzyme that controls all the key players in the metabolic pathways of endothelial activation and inflammation [93,94,95]. Although these pathways were not directly assessed in our study, future work could explore whether ALA supplementation modulates AMPK activity, lipid metabolism, and TGF-β/endoglin signalling in endothelial cells [86,87,96]. In the current study, ALA treatment not only increased endoglin expression and decreased IFN-γ concentration, while inducing a significant negative correlation between the two, but it also significantly reduced intracellular ROS production, corroborating ALA’s anti-inflammatory and antioxidative effect on ECs. 

As in the case of endoglin, IL-1α expression and activity depend on the present inflammatory signals. While ALA treatment reduces and down-regulates endotoxin-induced IL-1β, IL-6, and TNF-α production and expression in human cell lines and rats [97,98,99], evidence regarding effects on IL-1α is lacking. Nevertheless, IL-1α, described in the literature as an alarmin, is a central regulator of leukocyte-endothelial adhesion, inflammation, and endothelial activation, especially in CVDs [100,101]. It was reported by Brunn et al. (2014) that IL-1α, which is predominantly produced by ECs themselves, acts as an inflammation-limiting cytokine that supports wound healing at the site of injury [102]. It was also previously reported that in the event of infections, this cytokine can induce protective immune responses [103]. In the current study, a significant negative correlation was observed between ROS production and IL-1α concentration in the ALA-supplemented group. This topic clearly requires more thorough research, as we can only speculate on the dual functions of IL-1α and its regulation or activation by ALA. Although ALA’s protective effects are usually attributed to its conversion to EPA or DHA, this conversion is limited and inefficient. Increasing evidence suggests a direct role of ALA through its derived oxylipins [11,58,104,105,106].

Combined *n-3* PUFA supplementation mainly affected the expression of VCAM-1 and E-selectin, significantly increasing their levels. These results suggest a complex or possibly opposing interaction between the combined FAs in regulating CAM expression. For example, EPA supplementation led to a significant increase in the expression of all CAMs under moderate inflammation compared to the control group and/or baseline conditions. EPA produced a biphasic effect on ICAM-1 expression, significantly reducing it under baseline conditions, followed by an increase after endotoxin stimulation. This response highlights the context- and dose-dependent role of EPA in modulating endothelial inflammatory markers [107,108,109]. Under conditions of high inflammation, this effect was lost in the EPA group, and the expressions of VCAM-1, E-selectin, and Endoglin returned to near-baseline levels. Nevertheless, both EPA and combined *n-3* PUFA treatment effectively decreased pro-inflammatory cytokine and ROS production under inflammatory conditions, suggesting that their anti-inflammatory effects may occur through mechanisms that are at least partially independent of CAM regulation. Recent clinical evidence has highlighted important distinctions between the effects of EPA administered alone and in combination with DHA [30]. It was reported that EPA, when administered alone, has more beneficial effects than when combined with DHA in the context of cardiovascular outcomes [31]. The STRENGTH randomized clinical trial reported that a carboxylic acid formulation of EPA and DHA does not significantly improve outcomes of major cardiovascular events [32]. Distinct effects of different PUFAs on membrane structure, lipid oxidation, and the generation of pro-resolving lipid mediators require comprehensive evaluation. Such studies are needed to determine the influence on vascular health and their therapeutic and preventive potential.

A limitation of the present study is that the intracellular fatty acid composition following PUFA supplementation was not measured, and therefore, the extent of cellular incorporation remains to be determined. Further, NFκB p65 was only relatively quantified, lacking robustness but serving as a starting point for further investigation.

Overall, these results highlight distinct and molecule-specific effects of PUFAs on endothelial adhesion molecule expression, with DHA and ALA showing the most consistent anti-inflammatory profiles. The data also reveal that combined PUFA supplementation does not necessarily produce enhanced anti-inflammatory effects and may even upregulate the expression of certain adhesion molecules.

## 5. Conclusions

This study demonstrates that *n-3* PUFA supplementation exerts diverse, molecule- and dose-specific effects on endothelial cell function. While DHA and ALA consistently reduced oxidative stress and pro-inflammatory cytokine production, and downregulated key adhesion molecules such as ICAM-1 and E-selectin, EPA showed a biphasic response with initial ROS elevation followed by anti-inflammatory effects under high inflammation. Interestingly, ALA also modulated the expression of endoglin and IL-1α, suggesting a broader regulatory role in endothelial activation. Although combined *n-3* PUFA supplementation effectively reduced cytokine and ROS levels, it unexpectedly increased the expression of certain CAMs, indicating that simultaneous administration of multiple fatty acids may elicit complex or even opposing effects. These findings underline the importance of considering the specific type and context of *n-3* PUFA supplementation in strategies aimed at modulating vascular inflammation.

## Data Availability

The original contributions presented in this study are included in the article. Further inquiries can be directed to the corresponding authors.

## Supplementary Materials

The following supporting information can be downloaded at: https://www.mdpi.com/article/10.3390/biomedicines13112706/s1, Figure S1: Representative gating; Table S1: Correlation matrix *p*-values.

## Abbreviations

The following abbreviations are used in this manuscript:

AA	arachidonic acid
Ach	acetyl choline
ALA	alpha- linolenic acid
AMPK	AMP-activated protein kinase
ANOVA	analysis of variance
CAM	cell adhesion molecule
CRP	C reactive protein
CVD	cardiovascular disease
DCF-DA	2′,7′-dichlorodihydrofluorescein diacetate
DHA	docosahexaenoic acid
DHE	dihydroethidium
EC	endothelial cell
EDTA	ethylenediaminetetraacetic acid
ELISA	enzyme-linked immunosorbent assay
eNOS	endothelial nitric oxide synthase
EPA	eicosapentaenoic acid
FA	fatty acid
FBS	fetal bovine serum
FVD	fixable viability dye
FMD	flow-mediated dilation
GMFI	geometric mean fluorescence intensity
H_2_O_2_	hydrogen peroxide
HAEC	human aortic endothelial cell
HDL	high-density lipoprotein
HK-2	human kidney-2 cells
HFD-STZ	high-fat diet streptozocin rats
HUVEC	human umbilical vein endothelial cells
ICAM-1	intercellular cell adhesion molecule 1
IFNγ	interferon gamma
IL	interleukin
iNOS	inducible nitric oxide synthase
LA	linoleic acid
LDL	low-density lipoprotein
LPS	lipopolysaccharides
LSGS	low serum growth supplement
MAPK	mitogen-activated protein kinase
MCP-1	monocyte chemoattractant protein-1
NOS2	nitric oxide synthase 2
NOX	nicotinamide adenine dinucleotide phosphate oxidase
NFκB	nuclear factor kappa B
O_2_^−^	superoxide anion
OD	optical density
ONOO^−^	peroxynitrite
PBS	phosphate-buffered saline
PPAR	peroxisome proliferator-activated receptor
PUFA	polyunsaturated fatty acid
RANTES	regulated on activation, normal T cell expressed and secreted
ROS	reactive oxygen species
SHR	spontaneously hypertensive rat
SOD2	superoxide dismutase 2
sVCAM	soluble vascular cell adhesion molecule
TGFβ	transforming growth factor beta
TLR4	toll-like receptor 4
TNFα	tumor necrosis factor alpha
UCP3	uncoupling protein 3
VEGF	vascular endothelial growth factor
