# Supplementary material for: Polyunsaturated Fatty Acid (PUFA) Composition of Growth Medium Changes the Atherogenic Potential of Human Aortic Endothelial Cells (HAECs) Following Endotoxin Stimulation
Source: Biomedicines. 2025 Nov 4;13(11):2706. doi: 10.3390/biomedicines13112706 (PMC12650724; doi:10.3390/biomedicines13112706)
Supplement: Supplementary file 1 [file biomedicines-13-02706-s001.zip › Table S1 Correlation matrix p-values.pdf]

Spearman's correlation matrix (Benjamini-Hochberg correction)												
M200	Parameter	VCAM-1	ICAM-1	ENDOGLIN	E-SELEKTIN	TNFa	IL-6	IL-1a	IFNg	DCF-DA	DHE	NFkB
	VCAM-1	-	*0.001	0.466	*0.001	0.341	0.365	0.918	0.549	0.496	3.530	0.995
	ICAM-1	*0.001	-	0.644	0.060	0.651	0.668	0.616	0.778	0.662	0.755	0.727
	ENDOGLIN	0.408	0.564	-	0.894	*0.002	*<0.0001	0.184	0.202	*0.001	0.342	*0.030
	E-SELEKTIN	*0.001	0.060	0.894	-	0.800	0.756	0.707	0.107	0.870	0.931	0.777
	TNFa	0.205	0.521	*0.002	0.444	-	*0.001	0.428	0.478	*0.002	0.519	0.130
	IL-6	0.183	0.250	*<0.0001	0.588	*0.001	-	0.249	0.216	*<0.0001	0.108	*0.003
	IL-1a	0.408	0.821	0.553	0.808	0.856	0.499	-	0.922	0.790	0.749	0.749
	IFNg	0.235	0.556	0.252	0.213	0.558	0.303	0.307	-	0.491	0.109	0.854
	DCF-DA	0.165	0.221	*0.001	0.373	*0.001	*<0.0001	0.439	0.491	-	0.123	*0.001
	DHE	0.706	0.755	0.478	0.532	0.519	0.270	0.749	0.218	0.154	-	0.194
	NFkB	0.249	0.727	*0.04	0.666	0.130	*0.007	0.749	0.854	*0.005	0.117	-
ALA	Parameter	VCAM-1	ICAM-1	ENDOGLIN	E-SELEKTIN	TNFa	IL-6	IL-1a	IFNg	DCF-DA	DHE	NFkB
	VCAM-1	-	1.000	1.000	0.974	1.000	0.368	1.000	1.000	1.000	0.304	0.842
	ICAM-1	1.000	-	*0.040	*0.030	0.263	1.000	0.128	0.100	0.248	0.990	0.491
	ENDOGLIN	0.893	0.490	-	0.525	0.410	0.445	0.975	0.952	1.000	0.976	0.920
	E-SELEKTIN	0.974	*0.003	*0.030	-	0.273	1.000	0.056	0.164	0.081	0.905	0.998
	TNFa	0.910	0.490	0.525	0.410	-	0.445	0.975	0.952	1.000	0.976	0.920
	IL-6	0.368	1.000	0.974	1.000	0.556	-	0.719	1.000	0.923	0.164	1.000
	IL-1a	0.914	*0.043	0.096	0.056	0.975	0.431	-	0.428	*0.037	0.829	0.928
	IFNg	0.665	0.393	0.401	0.273	0.762	0.701	0.642	-	0.611	0.764	0.638
	DCF-DA	1.000	*0.009	0.310	0.108	0.834	0.659	*0.037	0.713	-	0.618	0.992
	DHE	0.152	0.417	0.990	0.905	0.325	0.164	0.932	0.637	0.618	-	0.890
	NFkB	1.000	1.000	1.000	1.000	1.000	0.948	1.000	1.000	0.992	1.000	-
EPA	Parameter	VCAM-1	ICAM-1	ENDOGLIN	E-SELEKTIN	TNFa	IL-6	IL-1a	IFNg	DCF-DA	DHE	NFkB
	VCAM-1	-	*0.007	*0.014	*0.001	0.791	*0.042	0.850	0.638	0.328	0.844	0.485
	ICAM-1	*<0.0001	-	*0.001	*0.002	0.909	*0.004	0.989	0.325	0.196	0.938	0.844
	ENDOGLIN	*0.004	*0.004	-	*0.002	0.878	0.072	0.760	0.350	0.083	0.775	0.790
	E-SELEKTIN	*0.004	*0.004	*0.002	-	0.882	*0.036	0.850	0.343	0.665	0.789	0.861
	TNFa	1.000	1.000	0.878	0.882	-	1.000	1.000	1.000	0.955	0.823	1.000
	IL-6	*0.014	*0.014	0.090	*0.048	0.678	-	0.214	0.245	0.393	0.345	0.309
	IL-1a	0.989	0.989	1.000	0.944	0.830	1.000	-	0.968	0.778	1.000	0.942
	IFNg	0.651	0.651	0.526	0.858	0.545	1.000	0.861	-	0.797	0.593	0.740
	DCF-DA	0.489	0.489	0.332	0.798	0.796	0.884	1.000	0.911	-	0.872	1.000
	DHE	0.938	0.938	0.997	1.000	0.823	1.000	1.000	0.988	0.997	-	1.000
	NFkB	1.000	1.000	0.922	0.984	0.841	1.000	0.707	1.000	1.000	1.000	-

	Parameter	**VCAM-1**	**ICAM-1**	**ENDOGLIN**	**E-SELEKTIN**	**TNFa**	**IL-6**	**IL-1a**	**IFNg**	**DCF-DA**	**DHE**	**NFkB**
**DHA**	**VCAM-1**	-	0.471	0.966	0.237	0.939	1.000	0.397	0.408	0.464	0.945	1.000
	**ICAM-1**	0.707	-	0.412	***0.001**	0.994	0.378	0.191	0.853	0.615	0.628	***0.035**
	**ENDOGLIN**	0.845	0.343	-	0.392	***0.045**	0.840	***0.029**	0.911	0.211	0.215	0.990
	**E-SELEKTIN**	0.711	***0.001**	0.490	-	0.805	0.674	0.118	0.713	0.834	0.572	0.607
	**TNFa**	0.939	0.994	***0.045**	0.940	-	1.000	0.410	1.000	0.737	0.823	1.000
	**IL-6**	0.748	0.756	0.945	1.000	0.917	-	1.000	1.000	0.903	0.935	0.124
	**IL-1a**	0.635	0.191	0.059	0.118	0.205	0.453	-	0.399	0.350	0.234	0.804
	**IFNg**	0.612	0.853	1.000	1.000	1.000	0.932	0.598	-	0.774	0.602	0.974
	**DCF-DA**	0.619	0.478	0.281	0.834	0.421	0.452	0.613	0.387	-	0.361	0.528
	**DHE**	0.945	0.717	0.322	0.686	0.686	0.935	0.390	0.301	0.361	-	1.000
	**NFkB**	1.000	0.070	0.990	0.758	1.000	0.062	1.000	1.000	0.879	1.000	-
**3PUFA**	Parameter	**VCAM-1**	**ICAM-1**	**ENDOGLIN**	**E-SELEKTIN**	**TNFa**	**IL-6**	**IL-1a**	**IFNg**	**DCF-DA**	**DHE**	**NFkB**
	**VCAM-1**	-	0.099	0.914	***0.045**	1.000	0.052	0.960	0.703	1.000	1.000	0.985
	**ICAM-1**	***0.033**	-	0.566	0.055	0.999	0.485	0.924	0.858	0.783	0.885	0.746
	**ENDOGLIN**	0.914	0.755	0.874	0.983	0.859	0.757	0.797	0.855	0.988	0.172	0.990
	**E-SELEKTIN**	***0.045**	0.055	0.874	-	0.708	0.100	0.874	0.627	0.755	0.826	0.641
	**TNFa**	0.938	1.000	1.000	1.000	-	1	1.000	0.958	1.000	1.000	1.000
	**IL-6**	***0.026**	0.364	1.000	0.100	1.000	-	1	0.087	1.000	1.000	0.941
	**IL-1a**	0.960	1.000	1.000	1.000	1.000	1.000	-	0.990	1.000	1.000	1.000
	**IFNg**	0.352	0.858	0.797	1.000	0.958	0.173	0.990	-	0.935	0.952	0.987
	**DCF-DA**	0.550	1.000	0.978	1.000	0.908	1.000	1.000	1.000	-	0.204	1.000
	**DHE**	0.815	1.000	1.000	1.000	0.868	1.000	1.000	0.952	0.204	-	0.891
	**NFkB**	0.591	0.932	0.172	1.000	0.635	0.941	0.748	1.000	1.000	0.990	-
