# Supplementary figures and images for: Polyunsaturated Fatty Acid (PUFA) Composition of Growth Medium Changes the Atherogenic Potential of Human Aortic Endothelial Cells (HAECs) Following Endotoxin Stimulation
Source: Biomedicines. 2025 Nov 4;13(11):2706. doi: 10.3390/biomedicines13112706 (PMC12650724; doi:10.3390/biomedicines13112706)

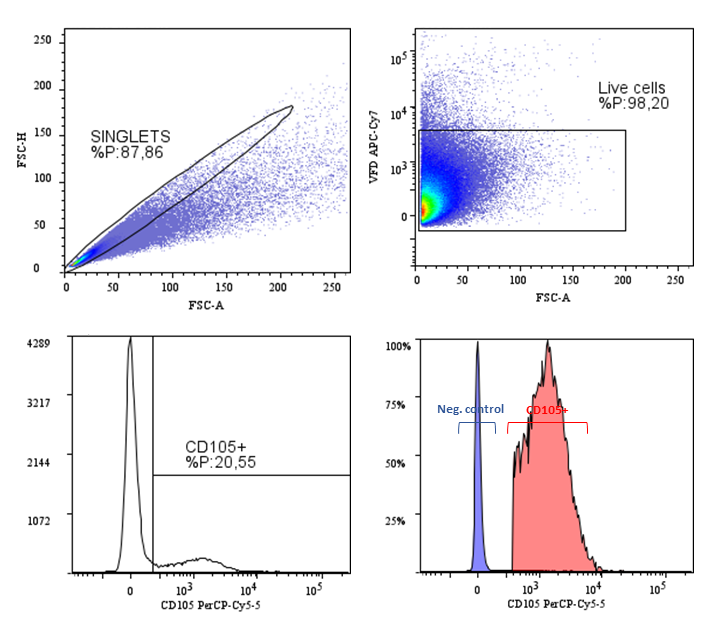

Supplement: Supplementary file 1 [file biomedicines-13-02706-s001.zip › Figure S1 Representative gating.png]
